# Combination of Mental Hospital Staffs

**Published:** 1913-12-13

**Authors:** 


### COMBINATION OF MENTAL HOSPITAL STAFFS.

Elsewhere in this issue we publish accounts of the meetings which were held last week at Manchester, Bristol, and London, following one at Birmingham, at which the assistant medical officers in mental hospitals met to discuss the possibility of combination as a remedy for the well-known grievances which have long been recognised among this class of mental hospital workers. These grievances—the absence of adequate salaries, the small chances of promotion, the virtual enforced celibacy through the rule which commonly insists that married assistant medical officers must live in while their wives must live out of the institutions—were at each meeting described by Dr. F. J. Stuart, senior assistant medical officer at Berrywood, Northampton, the honorary secretary of the new association, and the organiser of the new movement. His speeches were followed by discussions which took place in private, and the reason for this privacy throws a curious light upon the grievances of the service. The majority of the assistant medical officers, it seems, were unwilling publicly to take part in a discussion of their grievances through the fear that their expressions of opinion might prejudice their positions with the superintendents under whom they work. In certain cases, we understand, assistant medical officers were actually forbidden to attend, and in fact it seems that the meetings were often not looked upon with favour by those from whom leave had to be obtained. Dr. F. J. Stuart made it plain that into his own case such considerations did not enter, and that it was his freedom from restraining influences which enabled him to voice the grievances of his colleagues.

It is obvious, of course, that individual superintendents are not personally responsible for the existing discontent, and that, although a vicious system always encourages vicious administration, it is the system itself which is to blame. What that system is our readers for the most part know already, and can refresh their memories from the accounts of the meetings which appear on another page. It should be clear, however, before passing on to consider the general question of reform, that it is intolerable nowadays for any body of men, still more for members of any learned profession, to be forbidden to discuss the conditions of their work, or, at best, to have to discuss them in private for fear of prejudicing their position and prospects. In making clear that this unwarrantable check on discussion weighs upon assistant medical officers of mental hospitals, the new association has done good work, for such a check cannot bear the test of publicity. The moral, then, of the existing state of affairs is the need for combination among assistant medical officers. To drive this need home we have only to compare the discontent, fear of publicity and want of a means of common expression which have characterised the assistant medical staffs in mental hospitals hitherto, with the relative solidarity, official organ, and means of making themselves heard which are the happy possession of attendants. They have their own organisation, the Asylum Workers' Association, their own newspaper, and official recognition for their class. But these have only been won by combination. That must be the first step towards reform, and once it has been achieved ways will be found to remedy the small salaries and the enforced celibacy which are now justly complained of.

It is noteworthy that the only definite proposal that was made, in the meetings which were open to the press, for practically improving matters was to utilise the association at the triennial elections of mental hospital committees to bring public opinion to bear upon candidates to pledge themselves to vote for reforms, married quarters for the medical officers in particular. We confess that this does not seem to us a very statesmanlike proposal, but associations, like men, have to learn by practice, and that the suggestion was made indicates how little the true value of combination is understood by those who have had no experience of it. It is by the organising of pressure all along the line, by focussing the voice of the class organised constantly and everywhere, so that every committee feels that it is dealing with an organisation rather than with individuals, that success is won. An association can do much that an individual cannot, and though it cannot do all things, nor anything immediately, it can turn every event into an opportunity, and thereby raise the status of its members at every point.

